# Age, quality of life and mental well-being in adolescent population: a network model tree analysis

**DOI:** 10.1038/s41598-023-44493-w

**Published:** 2023-10-17

**Authors:** Leona Cilar Budler, Gregor Stiglic

**Affiliations:** Faculty of Health Sciences, University of Maribor, Zitna ulica 15, 2000 Maribor, Slovenia

**Keywords:** Physiology, Health care

## Abstract

This study presents the results of a network-based analysis of health related quality of life (HRQoL) among Slovenian adolescents. The study aimed to examine the relationship between HRQoL and mental well-being among adolescents of different age and gender groups. A cross-sectional study was conducted from November 2019 to January 2020 in 16 primary and 9 secondary schools in Slovenia. The KIDSCREEN-27 scale was used to collect the data on HRQoL, and the Warwick–Edinburgh Mental Well-being Scale to collect data on mental well-being. We used network model trees to demonstrate differences in psychometric network structure measuring correlations between different concepts in adolescent HRQoL. A total of 2972 students aged 10–19 years participated in the study. The significant split in the network tree (*p* < 0.001) indicated differences in relations between HRQoL subscale scores and mental well-being score among adolescents younger than 12 years old. In comparison to older adolescents the correlation between mental well-being and mood scores was significantly weaker in this group of the youngest participants (p < 0.001). A network model tree analysis also uncovered an interesting pattern based on gender and age (*p* < 0.013) where a correlation between mood and family support became weaker for female at the age of 12 and for male at the age of 16. Data mining techniques have recently been used by healthcare researchers and professionals. Network-based analysis is an innovative alternative to classical approaches in HRQoL research. In this study we demonstrate the significant differences in the perceptions of HRQoL and mental well-being among adolescents in different age and gender groups that were discovered using tree-based network analysis.

## Introduction

Adolescence is developmental stage marked by physical, psychological and social changes^1^. Adolescents are persons between the ages of 10 to 19^2^. This stage of development is associated with various risk factors that may influence adolescents’ mental health and well-being^1,3^. World Health Organization (WHO)^4^ defined mental health as “a state of well-being whereby individuals recognize their abilities, can cope with everyday stress, work productively and fruitfully, and make a contribution to their communities”. Concepts of mental health are the ability to realize intellectual and emotional potential, autonomy, competence, perceived self-efficacy, intergenerational dependence, and subjective well-being. Mental health is closely related to mental well-being (often named psychological or emotional well-being). The WHO^5^ defines mental well-being as “an individual’s ability to develop their potential, work productively and creatively, build strong and positive relationships with others and contribute to their community”. Mental well-being is a state of positive psychological and emotional health^6^. There is no universal definition of mental well-being, as this depends on the socio-cultural context of each individual. Mental well-being includes three concepts: satisfaction with life, hedonic well-being (positive and negative emotions), and eudemonic well-being (meaning in life)^7^. Stewart-Brown and Janmohamed^8^ argued that mental well-being is covering the hedonic perspective which is the subjective experience of happiness (affect) and life satisfaction, and the eudemonic perspective which includes positive psychological functioning, good relationships with others, and self-realization. Khan et al.^9^ stated that adolescents’ mental well-being is decreasing in last decades. The state of mental well-being is intricately linked to the broader concepts of well-being and quality of life. Sarriera and Bedin^10^ proposed a theoretical model of multidimensional well-being which includes subjective well-being, psychological well-being, psychosocial well-being and the socio-community well-being. They emphasize that well-being is a complex and multifaceted concept that has evolved over the last six decades and needs further investigation for a thorough understanding. Well-being serves as a foundational concept that includes various dimensions, including mental well-being. Mental well-being is integral to overall well-being and quality of life. Poor mental well-being can adversely affect one’s overall state of well-being and reduce the quality of life^11^. Quality of life is a more expansive term that includes well-being as one of its components. A high quality of life often implies good well-being, but it also includes other societal and environmental factors^11,12^. Twenge et al.^13^ agreed and added that mental well-being was lower in years when adolescents spent more time on screens (e.g., social media, the Internet, texting, gaming). Thus, effective intervention for promotion of adolescents’ mental well-being and prevention of mental disorders are needed^14^. Also, it is important to explore factors that influence adolescents’ mental well-being.

Adolescents’ mental well-being is closely related to their health-related quality of life (HRQoL), which is defined as “individuals’ functioning performance in life and their perceived well-being in physical, mental, and social domains of health”^15^. QoL is an indicator of overall well-being, happiness and satisfaction with life^16^. The concepts of well-being and HRQoL are complex and depend on the individual and the environmental context. Family, school, and peers are recognized as significant factors for successful development of adolescents^17^. It is known that parental support improves adolescents’ mental well-being^18^. Moreover, family and friends support were even more important during COVID-19 pandemic^19^. Study report by Ref.^20^ showed that the proportion of adolescents at risk for peer-relationship problems, pro-social behaviour problems and conduct behaviour is high. All concepts of QoL and mental well-being must be considered when interpreting support for adolescents’ mental well-being.

This study aims to observe the relationship between HRQoL and mental well-being among adolescents of different age and gender groups. Network model tree-based exploratory analysis with additional inferential statistical tests was used among Slovenian adolescents.

## Methods

### Study design

A cross-sectional study was conducted from November 2019 to January 2020 in 16 primary and 9 secondary schools in Slovenia.

### Settings and participants

The main criterion for inclusion in the study was the adolescent age between 10 and 19. The population was selected based on the systematic review, analysis, and synthesis of the literature. The research involved primary school (from the 5th to the 9th grade) and secondary school students (from the 1st to the 4th grade). Students who are under 10 years old and above 19 years old and those who are not included in the education system were excluded.

According to the Ref.^21^ there are 454 elementary and 182 secondary schools in Slovenia. Random sampling based on which the expected sample included all available students from 22 primary schools (5.0% of all elementary schools) and 12 secondary schools (5.0% of all secondary schools). Random sampling was chosen to represent student characteristics in the wider student population. The final number of participants was determined by the size of the total population of students, degree of confidence, and margin of error^22^, which amounted to 384 students. The sample size was increased to avoid the risk of attrition and dropout during the study, and to enable generalization of the findings. Survey questionnaires were distributed to 3860 students in primary and 3107 students from secondary schools who consent to participation in the classroom where the classes took place. The research involved primary school (from the 5th to the 9th grade) and secondary school students (from the 1st to the 4th grade). A total of 2972 students returned fulfilled questionnaires and participated in the study (Table 1).

**Table 1:** Sample characteristics.

	PS (*n* = 1489)	SS (*n* = 1483)
	M (SD)	M (SD)
Age	12.2 (1.5)	16.4 (1.3)

	*n* (%)	*n* (%)
Gender		
Female	768 (51.6%)	911 (66.8%)
Male	721 (48.4%)	452 (33.2%)
School year		
1st	0 (0.0%)	400 (29.3%)
2nd	0 (0.0%)	381 (28%)
3rd	0 (0.0%)	252 (18.5%)
4th	0 (0.0%)	311 (22.8%)
5th	264 (17.7%)	19 (1.4%)
6th	305 (20.5%)	0 (0.0%)
7th	266 (17.9%)	0 (0.0%)
8th	277 (18.6%)	0 (0.0%)
9th	377 (25.3%)	0 (0.0%)
Living environment		
With mother	1343 (90.7%)	1369 (91.9%)
With father	1204 (81.4%)	1205 (80.9%)
With sister/brother	1093 (73.9%)	1045 (70.2%)
With grandmother	316 (21.4%)	329 (22.1%)
With grandfather	214 (14.5%)	198 (13.3%)
Other	64 (4.3%)	59 (4.0%)

*PS*  primary school students, *SS* secondary school students, *M* mean, *SD* standard deviation, *n* number of participants.

### Instruments

The KIDSCREEN-27 measures physical well-being, mental well-being, autonomy, parental relationships, support and peer support, and the school environment. The questionnaire was developed under the KIDSCREEN-27 project^23,24^. It consists of five segments: physical activity and health, general well-being, and emotions about oneself, family and leisure, friends, school, and learning. Each item was scored on a five-point Likert scale ranging from 1 meaning “not at all” to 5 meaning “very much”. The KIDSCREEN-27 was validated in a pilot study among adolescents attending primary and secondary schools in Slovenia using a six-step analysis of the psychometric properties of the scale^25^ by the authors of this study.

The Warwick–Edinburgh Mental Wellbeing Scale (WEMWBS) questionnaire was developed in Scotland in 2006. The questionnaire includes 14 items and measures positive mental health and mental well-being over the past two weeks. When choosing the answers, a five-point Likert scale is offered and ranges from “none of the time” to “all of the time”. A translated questionnaire was used^26^. A sum of all answers gives a total WEMWBS score, which can be interpreted as poor (scores between 14 and 41), moderate (scores between 42 and 59), and excellent (scores above 60). The minimum score can be 14 and a maximum of 70^27^. The questionnaire was validated in a pilot study following the above-mentioned six-step analysis by Dima^25^. The Slovenian version of the WEMWBS achieved good validity and reliability in a sample of nursing students and can be recommended for future usage^28^.

The reliability of both instruments was assessed on the item-level using the Cronbach alpha measurements. Cronbach alpha levels for KIDSCREEN-27 subscales ranged from 0.778 to 0.869 with WEMWBS reaching a Cronbach alpha of 0.899. Tables 2 and 3 represent the Cronbach alpha values for scenarios of a single dropped item including mean and standard deviation values for all items in KIDSCREEN-27 and WEMWBS questionnaires.

**Table 2:** Cronbach alpha item-level analysis for KIDSCREEN-27.

	Alpha if item dropped	Mean	SD
Physical well-being (α = 0.778)			
General health	0.778	3.847	0.908
Feeling healthy	0.726	3.661	1.003
Physical activity	0.739	3.543	1.215
Ability to run	0.721	3.874	1.131
Energy	0.717	3.469	1.141
Psychological well-being (α = 0.849)			
Life	0.818	3.743	1.077
Good mood	0.822	3.611	1.024
Enjoy	0.828	3.643	1.106
Sad	0.831	3.824	1.177
Bad	0.833	4.006	1.255
Lonely	0.836	4.041	1.249
Happy	0.830	3.638	1.196
Autonomy and parent relation (α = 0.826)			
Free time	0.812	3.420	1.192
Free activities	0.817	3.236	1.242
Time parents	0.792	3.717	1.130
Parents fair	0.794	4.050	1.092
Parents talk	0.796	4.003	1.176
Money friends	0.806	3.766	1.214
Money expenses	0.804	4.043	1.151
Peers and social support (α = 0.869)			
Free time friends	0.866	3.797	1.143
Enjoy friends	0.800	4.169	1.079
Help friends	0.821	4.063	1.060
Reliability friends	0.842	4.043	1.101
School environment (α = 0.819)			
Happy school	0.790	3.193	1.180
Good school	0.749	3.500	1.099
Attention	0.766	3.516	1.072
Good relations	0.787	3.803	1.062

**Table 3:** Cronbach alpha item-level analysis for WEMWBS (α = 0.899).

	Alpha if item dropped	Mean	SD
I’ve been feeling optimistic about the future	0.896	3.580	0.989
I’ve been feeling useful	0.888	3.547	0.976
I’ve been feeling relaxed	0.891	3.604	0.987
I’ve been feeling interested in other people	0.899	3.670	0.971
I’ve had energy to spare	0.891	3.905	0.993
I’ve been dealing with problems well	0.891	3.534	0.997
I’ve been thinking clearly	0.893	3.766	0.928
I’ve been feeling good about myself	0.888	3.795	1.116
I’ve been feeling close to other people	0.895	3.520	1.053
I’ve been feeling confident	0.886	3.751	1.062
I’ve been able to make up my own mind about things	0.900	3.932	0.934
I’ve been feeling loved	0.892	3.753	1.065
I’ve been interested in new things	0.896	4.030	0.950
I’ve been feeling cheerful	0.888	3.864	0.926

Item-level alpha values for the scenario of dropping a single alpha value in the KIDSCREEN-27 ranged from 0.717 to 0.866. There were no cases where a removal of an item would improve the overall Cronbach alpha value of the subscale (Table 2). In case of WEMWBS scale the item-level alpha values in case of removing a single item ranged from 0.886 to 0.900. There was only one item where the removal would increase the overall alpha, but only by 0.001 (Table 3).

### Data analysis

Network model trees^29^ were used to demonstrate differences in psychometric network structure measuring correlations between different concepts in adolescent HRQoL. Initially, all participants with more than 50% of missing data were removed from the dataset. This step was followed by the data imputation using MissForest approach^30^ in the remaining sample. MissForest is an R package that implements the random forest imputation approach for missing data. MissForest first imputes all missing data using the mean/mode, and then fits a random forest on the present values and predicts the missing values for each variable with missing values. In this study, networks were built by splitting the sample into groups of participants by their gender and age to detect significant differences in the network structure allowing high level of model interpretability. All data analysis, including visualization of the results, was conducted using R statistical programming language^31^. Shapiro Wilk’s test was used to test the data for normality of distribution. Based on the results from the test of normality we used non-parametric Mann–Whitney test to check for the statistical significance of the differences.

### Ethical aspects

Before conducting the study, ethical approval was obtained from the Slovenian National Medical Ethics Committee (no. 0120-313/2019/13). The research was performed in accordance with relevant guidelines and regulations. Research has been performed in accordance with the Declaration of Helsinki. Before asking the permission to participate in study, parents and adolescents read research information form where study aims, rights and ethical aspect were explained. Adolescents and their parents gave their written permission to participate in this study.

## Results

A total of 2972 students aged 10–19 years participated in the study. A total of 768 (54.6%) females in primary school (PS) and 969 (66.6%) in secondary school (SS), and 638 (45.4%) males in primary and 487 (33.4%) in secondary school participated in the study. Median age of students in primary school was 12 (IQR = 2) and 16 (IQR = 2) for students in secondary school. Other sample characteristics are shown in the Table 1.

**Figure 1:**
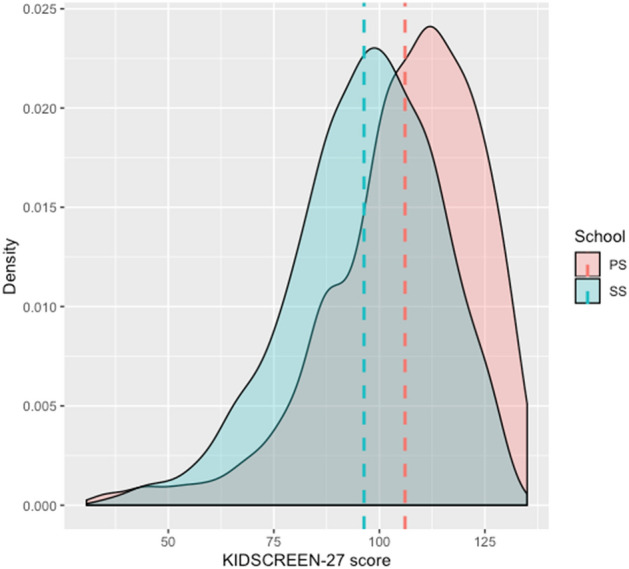
The KIDSCREEN-27 score distribution. *PS* primary school students, *SS* secondary school students.

**Figure 2:**
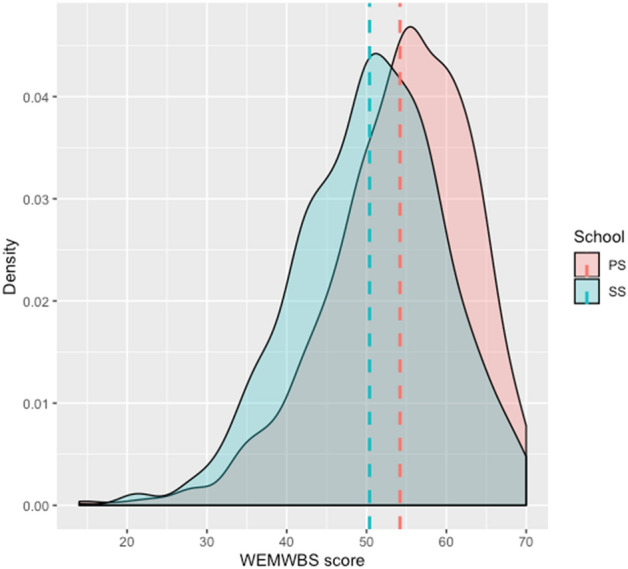
The WEMWBS score distribution. *PS* primary school students, *SS* secondary school students.

To conduct further analyses, data distribution for the KIDSCREEN-27 (Fig. 1) and the WEMWBS (Fig. 2) scale by primary and secondary school was explored. We also conducted statistical tests of normality (Shapiro–Wilk’s) where the deviation from the normal distribution was confirmed for KIDSCREEN-27 (p_PS_ < 0.001, p_SS_ < 0.001) and WEMWBS (p_PS_ < 0.001, p_SS_ < 0.001) data. We also calculated skewness of the distribution for all six variables which ranged from − 0.396 to − 1.258. Additionally, we calculated kurtosis which ranged from 2.627 to 4.415. Therefore, we used nonparametric tests in statistical testing of the findings from the exploratory analysis.

As presented in Fig. 2, data is distributed normally. There is also an evident difference in the mean values of mental well-being and HRQoL between primary school students and secondary school students. It is evident that primary school students have higher HRQoL and better mental well-being than secondary school students.

Median value of the mental well-being of primary school students was 55 (*IQR* = 12) and median value among secondary school students was 51 (*IQR* = 13), showing that younger students have better mental well-being than older students which was also confirmed by a Mann–Whitney test (p < 0.001).

**Figure 3:**
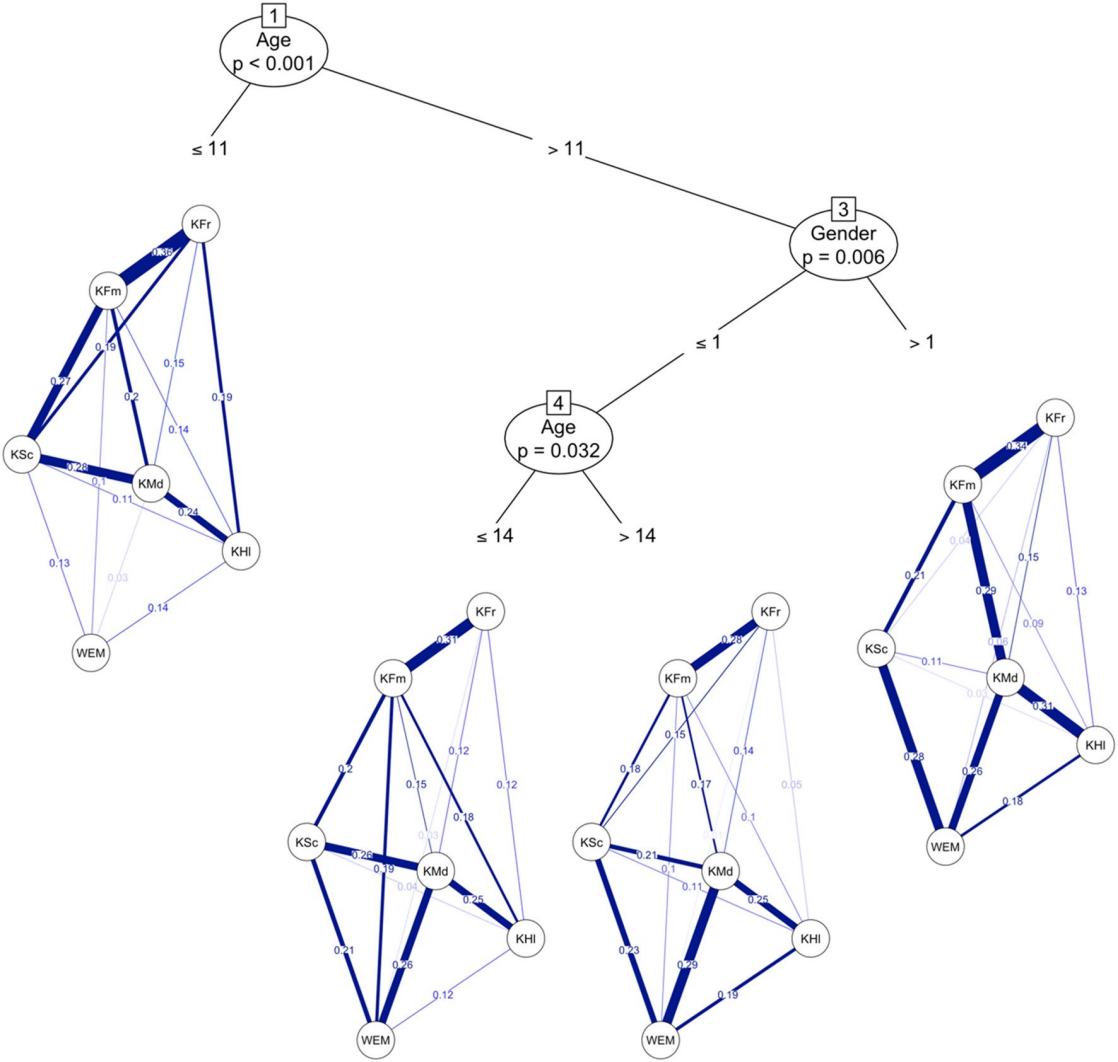
Network model tree comparing network structure based on gender and age. *WEM* Warwick Edinburgh mental well-being scale, *KFr* social support and peers subscale, *KFm* parents relations & autonomy subscale, *KSc* School environment subscale, *KMd* Psychological well-being subscale, *KHI* Physical well-being subscale.

Moreover, using network model trees we demonstrate the differences in psychometric network structure measuring correlations between different concepts in adolescent HRQoL. The first significant split in the network tree (*p* < 0.001) indicated differences in relations between HRQoL subscale scores and mental well-being score among students younger than 12 years old (Fig. 3). In comparison to all other groups the correlation between mental well-being and mood scores was significantly weaker in this group of the youngest participants. This might suggest that the WEMWBS might not be the most appropriate tool to measure mental well-being in the young adolescent population. A network model tree analysis also uncovered an interesting pattern based on gender and age (*p* < 0.013) where a correlation between mood and family support became weaker for female at the age of 12 and for male at the age of 16. This might correspond to puberty related changes in adolescence starting earlier in girls compared to boys which can be supported by the literature.

**Figure 4:**
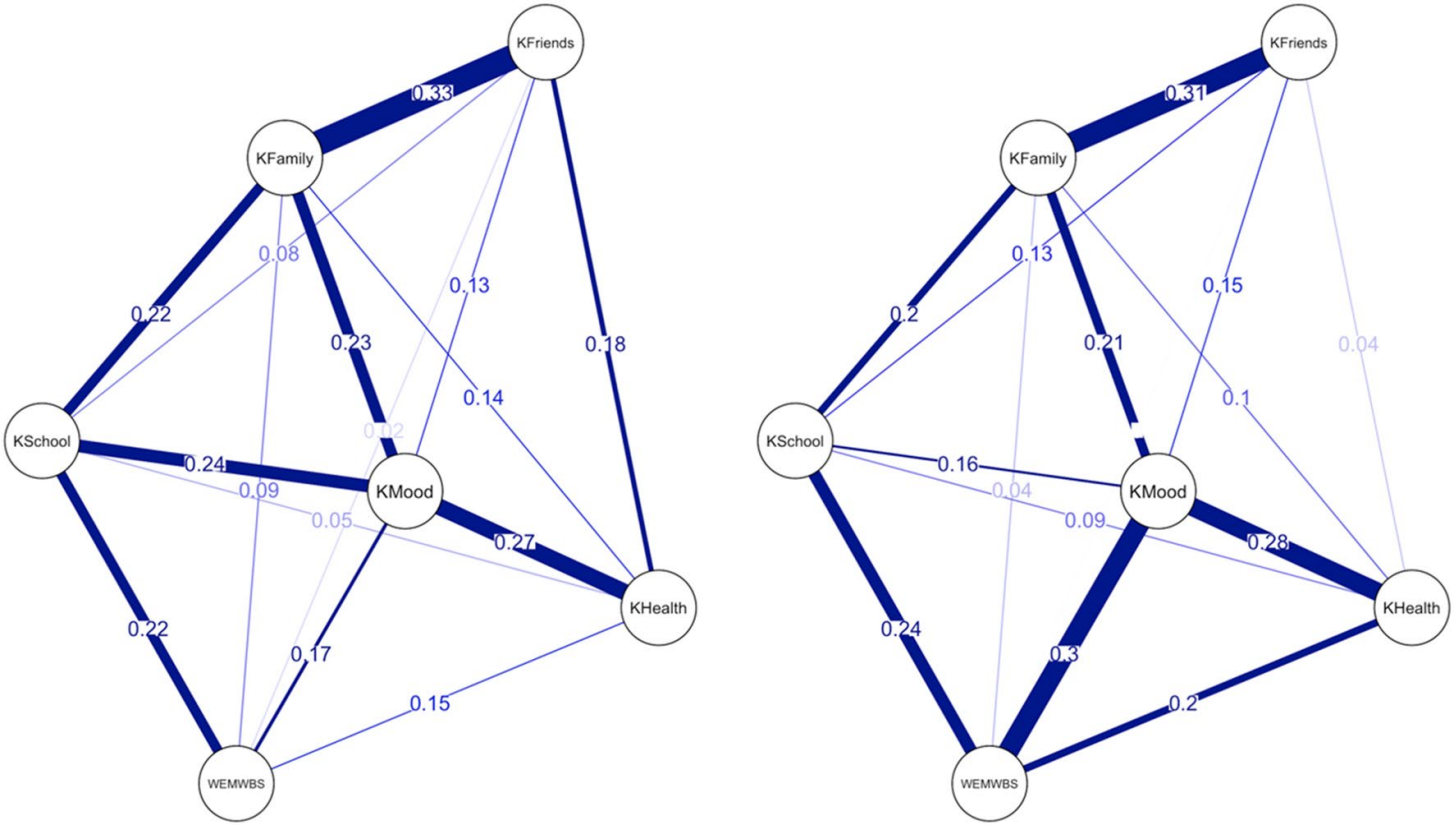
Correlations between HRQoL concepts for younger (left) vs. older (right) adolescents. *WEMWBS* Warwick Edinburgh mental well-being scale, *KFriends* Social support and peers subscale, *KFamily* Parents relations & autonomy subscale, *KSchool* School environment subscale, *KMood* Psychological well-being subscale, *KHealth* Physical well-being subscale.

Correlation between HRQoL concepts differs among younger and older adolescents. Among younger adolescents correlations are strong between mood and general health (*r* = 0.27, p < 0.001), mood and support from school (*r* = 0.24, p < 0.001), and family (*r* = 0.23, p < 0.001). On the other hand, among older adolescents correlations are strong between mood and mental well-being (*r* = 0.30, p < 0.001), mood and general health (*r* = 0.28, p < 0.001) and family support (*r* = 0.21, p < 0.001). Older adolescents’ mental well-being is strongly correlated with their general health (*r* = 0.20, p < 0.001), mood (*r* = 0.30, p < 0.001), and school support (*r* = 0.24, p < 0.001) (Fig. 4). Due to relatively low correlation coefficients, we also performed caclualtion of some centrality measures in the network.

## Discussion

The link between adolescents’ mental well-being and quality of life is now known in the literature and research into these dimensions is essential for the development of preventive and promotional measures aimed at creating a foundation for good mental health in adulthood^32^. Poor adolescents’ mental well-being can decrease their satisfaction with life and can lead to various mental health problems in adulthood^33^. Although the correlation between adolescents’ mental well-being and quality of life is known, there is a gap in explanations of those correlations. It is not known which factors have influence on the mental well-being of adolescents in correlation with their HRQoL.

Main findings of this study show differences in relations between KIDSCREEN-27 subscale scores and WEMWBS scores among adolescents younger than 12 years old. This correlation has not been yet explored; thus, results cannot be directly compared to other studies. The correlation between mental well-being and psychological well-being was significantly weaker in group of the youngest participants. It is also evident that correlation between psychological well-being and family support became weaker for female at the age of 12 and for male at the age of 16. It is known that family support has the biggest role in maintaining adolescents’ mental well-being in younger adolescents. While friends and peers have bigger role in older adolescents.

Giannakopoulos et al.^34^ found out that mental health of parents is correlated with adolescents’ better mental well-being, moods, and emotions. Also, adolescents’ male gender, younger age, absence of chronic health care needs, high social support, and higher family income were positively associated with better HRQoL. Similar to our results, Meade and Dowswell^1^ reported lower scores in HRQoL among female students where scores also declined over time across two of the five HRQoL dimensions (social support and peers, and school environment). Age differences were found across all but one dimension (autonomy and parents relations). HRQoL, the teacher’s opinion of performance and the perception of health status are better among adolescents with better family support^35^. When caring about adolescents mental well-being, both social support and quality of life should be considered. Future research should be focused on exploring other factors that may influence adolescents mental well-being.

### Limitations

Although, this study revealed interesting and new findings, there are a few limitations which should be considered when interpreting study results. First, a convenience sample was used and the generalization of the findings to the entire population of adolescents might be limited. Also, the WEMWBS and the KIDSCREEN-27 are self-reporting scles which may lead to different biases and limitations. Participants may give socially acceptable answers. They may not be able to assess themselves accurately. For younger adolescents, the wording of the questions may be confusing or have different meaning. Also, they may not understand the concept of mental well-being. From the technical perspective one should be aware that decision tree splits always introduce a certain degree of instability. In practice this could mean that a small change in the data could produce a different tree and consequently different networks^25^. However, this problem can be mitigated using a large sample size in most cases. It should also be noted that although statistically significant the correlation coefficients are relatively small. In such cases additional network analysis metrics might be used. However, due to fully connected unweighted networks used in our approach this would not provide any additional information on strength of connections. On the other hand, a large sample of the data should alleviate some of the concerns in this regard. Additionally, it should be noted that for an exploratory study, it would be very useful to obtain information about the potential cofounders such as socio-economic status, health, and diseased conditions, or early life stress.

## Conclusions

The correlation between adolescents’ mental well-being and their HRQoL is weaker among the youngest participants. This might suggest that the WEMWBS scale is not the most appropriate tool to measure mental well-being in the young adolescent population. Also, concept of mental well-being is probably not understood among young adolescents. This should be further explored and researched. The correlation between mood and family support is weaker for female at the age of 12 and for male at the age of 16. This might be explained with puberty related changes in adolescence starting earlier in girls compared to boys. The average age for girls to begin puberty is 11, while for boys the average age to begin puberty is 12 or later. Those differences in puberty changes between different genders in correlation with their mental well-being is known but poorly understood and researched. All important persons should be involved in decisions about adolescents’ care to provide high-quality and person-directed care. Also, it is important to involve nurses in schools to ensure holistic and continuous care for adolescents who need help and may develop mental health problems. More practical options for adolescents and parents must be provided to get the needed help when faced with mental health issues. Healthcare and educational institutions could ensure professionals for adolescents to help them if they struggle with financial problems, poverty, bad interpersonal relationships, social exclusion, troubles with learning, or other factors that may contribute to worsening mental well-being and mental health.

## Data Availability

The datasets used and analysed during the current study available from the corresponding author on reasonable request.
